# Therapy Settings Associated with Optimal Outcomes for t:slim X2 with Control-IQ Technology in Real-World Clinical Care

**DOI:** 10.1089/dia.2023.0308

**Published:** 2023-11-23

**Authors:** Laurel H. Messer, Marc D. Breton

**Affiliations:** ^1^ Tandem Diabetes Care, San Diego, California, USA.; ^2^ Center for Diabetes Technology, University of Virginia, Charlottesville, Virginia, USA.

**Keywords:** Automated insulin delivery, Insulin pump, Type 1 diabetes, Diabetes technology, Hybrid closed loop

## Abstract

**Objective:**

To determine insulin dosing parameters that are associated with and predict optimal outcomes for people using t:slim X2 with Control-IQ technology (CIQ).

**Methods:**

Retrospective deidentified data from CIQ users were analyzed to determine the effect of Correction Factor, Carbohydrate-to-Insulin (C:I) Ratio, and basal rate settings (standardized by total daily insulin [TDI]) on glycemic control. We performed an associative analysis followed by linear regressions to determine the relative importance of the settings and confounding variables (e.g., age or number of user-initiated boluses) in predicting consensus glycemic outcomes.

**Results:**

Data from 20,764 individuals were analyzed (median age 39 years [interquartile range 19, 59], 55% female, TDI 46.4 U [33–65.2]). More aggressive Correction Factor settings, C:I ratio settings, and basal programs were all strongly associated with higher time in range (TIR, 70–180 mg/dL) and to a lesser degree to higher time <70 mg/dL. In linear regression, more aggressive Correction Factor predicted higher TIR, lower coefficient of variation, and importantly had only negligible impact on time below range. Higher basal rate settings and lower C:I ratio predicted increased TIR as well as increased hypoglycemia. The most important predictor in all glycemic outcomes was the average number of user-given boluses per day.

**Conclusion:**

Basal rates, C:I ratios, and Correction Factor settings all impact glycemic outcomes in CIQ users in usual clinical care. The correction Factor setting may be the most impactful “lever to pull” for clinicians and CIQ users to optimize TIR while not increasing hypoglycemia.

## Highlights

- We analyzed real-world evidence to see if there are parameter settings associated with better glycemic outcomes for users of Control-IQ technology (advanced hybrid closed-loop).
- More aggressive basal rates, carbohydrate-to-insulin ratio, and Correction Factor were all associated with higher time in range 70–180 mg/dL, although also slightly increased hypoglycemia.
- In predictive analysis, more aggressive Correction Factor predicted higher time in range, lower variability, and only negligible impact on hypoglycemia
- While all settings are useful with Control-IQ technology, the Correction Factor may be the most impactful “lever to pull” to optimize outcomes

## Introduction

Automated insulin delivery (AID) has decisively changed the diabetes care landscape for people who require intensive insulin therapy. AID systems consist of an insulin pump for continuous subcutaneous insulin infusion, a continuous glucose monitor to measure interstitial glucose levels every 5 min, and proprietary algorithms to determine automated insulin dosing requirements based on glucose levels and prior insulin delivery. According to meta-analyses, AID has shown to improve time in range (TIR, 70–180 mg/dL) an average of 9%–12% compared to other intensive insulin regimens, without an increase in hypoglycemia.^1,2^ Currently, the commercially available systems in the United States include the Tandem t:slim X2 with Control-IQ technology (CIQ), Minimed 670G/770G and 780G, Insulet Omnipod 5, and the iLet Bionic Pancreas. A recent meta-analysis of the randomized clinical trial outcomes in people ages 2–72 years using CIQ show an 11.5% adjusted group improvement in TIR compared to standard of care with Continuous Glucose Monitoring (CGM).^3^

The Control-IQ system is an advanced hybrid closed loop that automates insulin delivery, aiming for glucose levels of 112.5–160 mg/dL predicted 30 min into the future.^4–6^ The system uses basal rates, Correction Factor (also known as insulin sensitivity factor), and carbohydrate to insulin (C:I) ratio entered by the user as part of their algorithm inputs, whereas the correction target and active insulin time are fixed in the system and cannot be changed when automation is turned on (110 mg/dL target and 5 h insulin-on-board). The Control-IQ algorithm dynamically adjusts basal insulin doses every 5 min to achieve predicted glucose levels, and administer an automatic correction dose up to once per hour if glucose levels are predicted to rise above 180 mg/dL. This autocorrection dose is calculated based on the future predicted glucose and delivered at 60% of the Correction Factor dose.

In clinical practice, health care providers (HCPs) initiate AID systems and traditional insulin pumps with the same settings, even though it is unknown if this is the best approach for AID. These settings are generally in line with the American Association of Clinical Endocrinologists/American College of Endocrinology Insulin Pump Management Taskforce consensus guidelines published in 2014.^7,8^ Guidelines suggest programming a basal rate at ∼50% of total daily insulin (TDI) divided by 24 h. The Correction Factors are calculated by dividing 1700 by TDI (“1700 rule”),^9^ and C:I ratios by dividing 450 by the TDI (“450 rule”).

While these settings appear to be safe starting parameters for AID, the best approach for optimizing these settings is not known. It is an especially difficult conundrum considering that each AID has distinct parameters that affect automatic dosing,^10^ meaning that settings adjustments that work for one system may not work for another AID system. Therefore, it is important to inform HCPs and people with diabetes *with system specific information* for how to initialize and tune AID systems' settings for optimal glycemic outcomes. Therefore, the purpose of this analysis is to determine which parameters are associated with optimal glycemia to empower users to optimize system settings.

## Research Design and Methods

We performed a retrospective analysis of data from CIQ users in the United States who had descriptive data available in the Tandem Diabetes Care Customer Relations Management database, as well as pump and glycemic data uploaded to the t:connect Web application (either through the Tandem Diabetes Care t:connect Uploader or through the mobile app). Participants consented to the use of their data for research purposes as part of their onboarding to Tandem Diabetes Care in initiating a t:connect account. The data were deidentified, and no IRB approval was sought for this retrospective analysis.

We included previously collected data from individuals with a diagnosis of type 1 diabetes and had a minimum of 21 days of data in the month before using CIQ and 60 days out of the 3 months afterward as of January 2023. Of these, day-level data were excluded if they included <80% CGM values, <50% Control-IQ use, or <1 U of insulin delivery (indicating non-use). All variables were averaged for each remaining subject across all remaining days.

Descriptive data included type of diabetes, age, body weight at database enrollment, and gender. Insulin delivery data included the total number of insulin units delivered each day, the number of daily user-initiated boluses, as well as the 24 h averages for pump profiles: basal, C:I ratios, and Correction Factors. CGM data included daily averages of consensus outcomes, including TIR 70–180 mg/dL, time below range <70 mg/dL, time below range <54 mg/dL, and coefficient of variation.^11^

### Analysis plan

To analyze the relationship between pump settings and glycemic outcomes, we used a two-prong approach: First, we explored the association between basal rates, Correction Factor, C:I ratio parameter settings by quartile and key glycemic outcomes. Second, we performed a linear regression to quantify the amplitude and importance of each parameter as predictors for individual glycemic outcomes.

Pump settings were standardized by TDI for comparison. Daily average Correction Factor and C:I ratio values were multiplied by the participant's average TDI to standardize across subjects and quantify what TDI based “rule” was used by the participant (Correction Factor therefore become a number between 500 and 6000, C:I a number between 100 and 1000). Daily average basal rates obtained from the pump settings were first multiplied by 24 and then divided by TDI, creating an index representing the expected basal/bolus balance based on the basal settings (e.g., from 10% to 90% of TDI).

It is important to note, however, that these do not reflect the basal amounts actually delivered. Since Control-IQ modulates basal rates every 5 min as part of its AID algorithm, the user may receive more or less than this ratio. Therefore, this calculation is interpreted as how aggressively the basal program was set in the insulin pump. For Correction Factor, C:I ratios, and basal parameters, we extracted median and quartiles to form four groups of participants of approximately equal size for each setting.

The standardized pump settings for Correction Factor, C:I ratios, and basal were each divided into quartile groups, and glycemic outcomes reported per quartile. Next, the settings were further divided into smaller bins on unequal sizes to analyze trends of glycemic outcomes: For Correction Factor, 1200 to 2200/TDI by increments of 100/TDI, C:I ratios 150 to 650/TDI by increments of 50/TDI, and basal as percent TDI from 25% to 75% by increments of 5%.

Finally, we performed a linear regression model to analyze the effect, amplitude, and importance of each pump parameter on the observed glycemic outcomes. The dependent variables were chosen per the consensus CGM outcomes^11^ and the predictors included age (years), number of user-given boluses per day, relative TDI (U/kg), C:I ratio TDI index, Correction Factor TDI index, and Basal program TDI fraction.

To avoid issues with outliers biasing the linear regression, the first and last percentiles of each predictor were removed. Standardized coefficients and “importance” (standardized change in model *R*^2^ if variable is last entered^12^) were used to quantify how impactful each predictor may be relative to others, while regression coefficients were used to quantify the amplitude of changes in glycemic outcomes associated with changes in parameter settings. Significance threshold for *P*-values was <0.05, adjusted for multiplicity using Bonferroni correction.^13^ Statistical analysis was performed using SPSS 28.0.1.1 (IBM Corp.).

## Results

In total, 2,607,812 days of data were reviewed from 23,141 individuals. Of these, data from 20,764 individuals met criteria and were included in analysis (Table 1).

**Table 1.** Sample Demographics of the 20,762 Control-IQ Users Included in Analysis

	Median [IQR]	Min–max
Age	39 [19–59]	1–93
Gender	55% female 45% male <1% other	—
Body weight [kg]	74.6 [61.0–89.5]	24.9–139.5
No. of available days	80 [69–86]	14–91
Percent time in range 70–180 [%]	71.5 [62.3–79.6]	8.0–99.9
Percent time below 54 mg/dL [%]	0.1 [0.05–0.3]	0–8.5
Percent time below 70 mg/dL [%]	1 [0.5–1.9]	0–19.6
Percent time above 180 mg/dL [%]	27.1 [18.7–36.6]	0–92.1
Percent time above 250 mg/dL [%]	5.6 [2.5–10.9]	0–63.01
Mean CGM [mg/dL]	155.5 [143.6–169.6]	91.8–279.2
Coefficient of variation CGM [%]	29.7 [26.7–32.8]	11.2–46.9
Total daily insulin [U]	46.4 [33–65.2]	5.5–282.1
Total daily insulin per body weight [U/kg]	0.6 [0.5–0.8]	0.1–3.4
Average C:I ratio [g/U]	9 [6.5–12.2]	0–155.7
Average correction factor [mg/(dL·U)]	42.7 [30–57.2]	0.5–465.6
Average basal rate [U/h]	0.9 [0.6–1.3]	0.1–6.2
No. user-initiated boluses/day	5.2 [3.8–6.8]	0–28.9

CGM, Continuous Glucose Monitoring; C:I, carbohydrate-to-insulin; IQR, interquartile range.

**FIG. 1.**
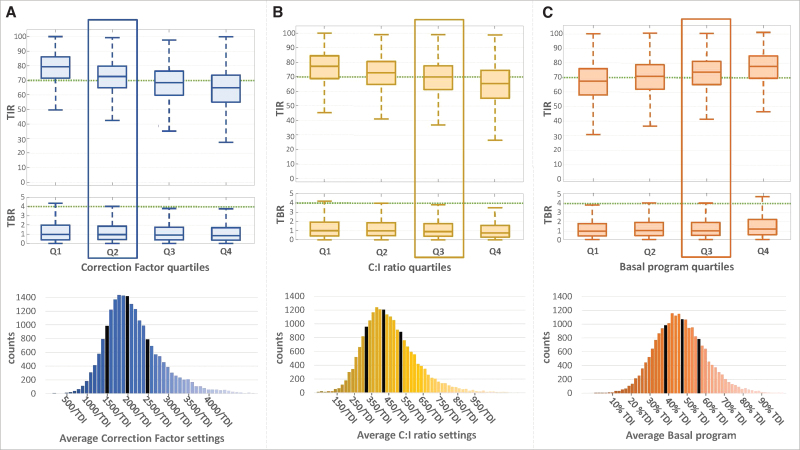
**(A)** Distribution (box and whiskers plot) of TIR (70–180 mg/dL, upper graph) and time below range (<70 mg/dL, middle graph) by quartiles of Correction Factor TDI-based index settings, with box highlighting the quartile containing the AACE guideline index (1700/TDI). Bottom graph presents distribution of the Correction Factor TDI-based index with quartile limits in black. **(B)** Similar graph for the C:I ratio TDI-based index, with AACE guideline of 450/TDI highlighted. **(C)** Similar graph for the Basal TDI-based index with AACE guideline of 50% basal highlighted. C:I, carbohydrate-to-insulin; TBR, Time Below Range; TDI, total daily insulin; TIR, time in range.

The Correction Factor settings, C:I ratio settings, and basal program were all clearly associated with differences in CGM-derived glycemic control metrics (Fig. 1 below, Supplementary Tables S1 and S2 in Supplementary Material). For the Correction Factor settings, there was a + 14% median TIR difference (79.1% vs. 65.0%, *P* < 0.001) between the most aggressive and least aggressive Correction Factor quartile (respectively left and right in Fig. 1A). The C:I ratio settings showed a similar effect with +11% difference in median TIR between the least aggressive and most aggressive quartiles (77.0% vs. 65.6%, *P* < 0.001, left to right, Fig. 1B). The basal program indexed as percent of TDI showed a + 10% median TIR between the most aggressive and least aggressive basal program (76.4% vs. 66.4%, *right to left*, respectively, *P* < 0.001).

While all parameter settings showed similar results for other glucose metrics (Supplementary Table S1), the results differ slightly for time below range. For time below 70%, the parameter changes across quartiles were associated with a + 0.15% change for Correction Factor, +0.23% change for C:I ratio, and +0.25% change for basal programs, which are not likely to be considered clinically significant across the population. Time <54 mg/dL was largely insensitive to changes, not differing >0.03% across different parameters.

**FIG. 2.**
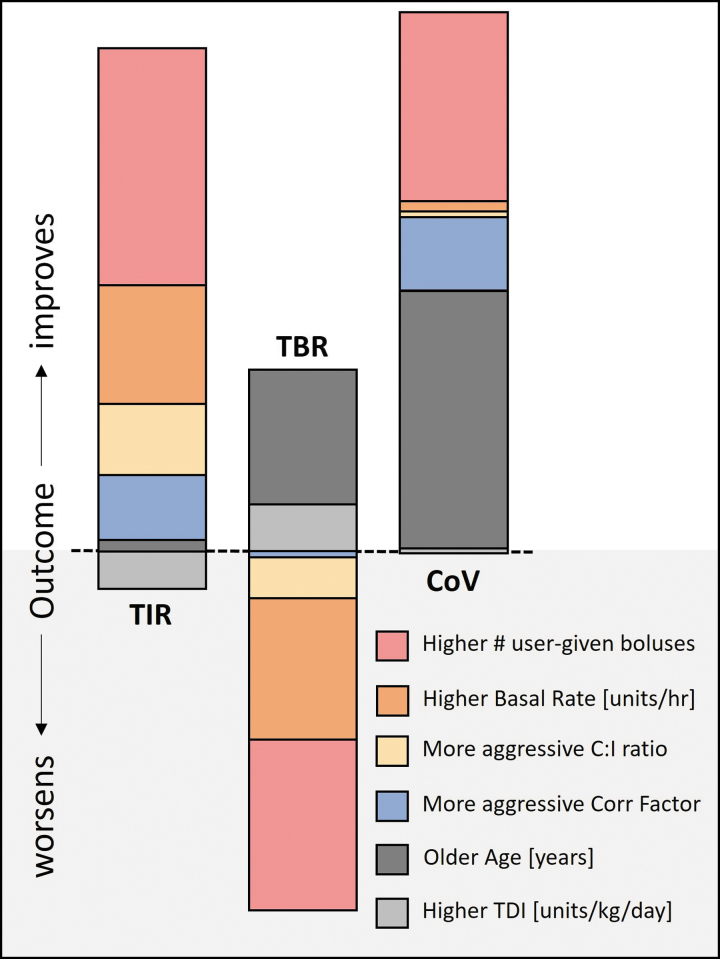
Relative importance (size of bar) of key variables predicting TIR 70–180 mg/dL, time below range 70 mg/dL, and the CGM CoV, with added directionality for a better (higher TIR, lower TBR, lower CoV) or worse outcome. CGM, Continuous Glucose Monitoring; CoV, coefficient of variation.

The linear regression allowed us to assess the importance of the programmed settings as well as bolus behavior, age, and TDI on predicting glycemic outcomes (Fig. 2 and Supplementary Table S3). The observed correlations between predictors were low to negligible. Together, these predictors explained a substantial amount of the variance in TIR (*R*^2^ = 42.4), however, only 3.6% of time below range. For the coefficient of variation, 20.8% of the variance was explained by these predictors.

Figure 2 indicates the relative importance of each predictor and the directionality of the prediction on TIR, time below range, and coefficient of variation. The most important predictor in all outcomes is the number of user-given bolus per day, improving TIR but contributing to more hypoglycemia. For the pump settings of interest, higher basal rate settings are the most important contributor to increased TIR, followed by more aggressive Correction Factor and C:I ratios. For time below range <70 mg/dL, high basal rate settings made the most impact on increasing hypoglycemia, followed by aggressive C:I ratios. The Correction Factor was not substantially important in predicting hypoglycemia. For coefficient of variation, a more aggressive Correction Factor predicted improvement in the value, with much smaller effects from basal rate setting and C:I ratio.

## Discussion

This is the first analysis of glycemic outcomes associated with specific Control-IQ settings in a large cohort of over 20,000 users. In descriptive analysis, a more aggressive Correction Factor, C:I ratio, and basal rate were all associated with higher TIR. These observations were confirmed using linear regression, where more aggressive basal rates, Correction Factor, and C:I ratios all predicted higher TIR. Interestingly, while more aggressive C:I ratio and basal were clearly associated with increased Time Below Range (however small), that was not the case for the Correction Factor. Furthermore, a more aggressive Correction Factor predicted improved coefficient of variation. Taken together, this analysis indicates important clinical insights for adjustment of Control-IQ settings for optimal glycemia.

The performance of CIQ is impacted by programmable Correction Factor, C:I ratio, and basal rates, and this analysis indicates that the Correction Factor may be the most important parameter for maximizing TIR while minimizing change in hypoglycemia. In this sample, 67% of the Control-IQ users had less aggressive settings than set forth by the AACE guidelines (1700/TDI). Since there is no prior research indicating the particular importance of Correction Factor to the Control-IQ system, this is not surprising. The Correction Factor is a unique parameter with CIQ, in that it affects user-programmed boluses, the autocorrection bolus administered up to once hourly (calculated on predicted glucose, administered as 60% of calculation dose), and the intensity of basal modulation.

The autocorrection feature has been shown to meaningfully impact TIR for users who administer fewer boluses, which makes it a useful parameter for CIQ users regardless of bolusing behavior.^3,14,15^ It is worth noting that higher Correction Factor was the least likely to be associated with higher time below 70 mg/dL. This may be explained by the fact that the Control-IQ algorithm's dynamic basal modulation blunts the risk of hypoglycemia by effectively reducing the basal delivery in the hours after bolus insulin is administered.

The C:I ratios are a well-known tunable parameter for AID systems,^10,16^ with consensus guidance suggesting more aggressive settings with AID initiation.^17^ It is therefore not surprising that 54% had C:I ratios that were more aggressive than the AACE guidelines (450/TDI). The assumption that more aggressive C:I ratios are useful in AID are confirmed in this analysis, both in association analysis as well as linear regression. With Correction Factor and C:I ratios both primarily affecting bolus settings, it stands to reason that strengthening both settings has a similar effect on TIR.

This report provides much needed insight for HCPs and CIQ users to consider for maximizing TIR. There are few existing resources for HCPs indicating best ways to optimize AID settings, which is why traditional insulin pump dosing guidelines are often still used.^8^ Further, other reports have suggested optimal settings for different AID systems that cannot be applied to CIQ due to differing tunable parameters.^10,18,19^ An implication of this report is that, for Control-IQ users, strengthening the Correction Factor may improve TIR without necessarily impacting hypoglycemia. Both C:I ratio and basal rate changes can be considered as well, although overall basal increase may predict more hypoglycemia beyond 55% of TDI.

As with all insulin dose adjustments, safety should be maximized by making small adjustments at a time (10%–20%), and reevaluating after a few days (except in the case of marked hypoglycemia increase, in which case settings should be adjusted more imminently). Future work may elucidate if there is more specific guidance that can be offered to individual users based on bolusing behavior patterns, and use of sleep activity.

The strengths of this article include the analysis of a very large (23,000) population of CIQ users in the real world across a large range of ages, weights, TDI requirements, number of daily boluses, and glycemic outcomes. The data are novel and lead to key clinical insights. This analysis has limitations as well. Data were analyzed for association and linear regression prediction but did not include a temporal aspect. This means that there can be no definitive causal link drawn between CIQ settings and glycemic outcomes. While our sample size was large, there was not a large representation of children and adolescents, and no information on ethnicity and race, which raises the question of whether these results apply across different racial/ethnic groups and youth.

Time in the hypoglycemic range was lower in this data set than in prior clinical trial data, and time below 54 mg/dL was particularly so, limiting our capacity to observe the impact of different settings on these outcomes. Finally, it is important to note that people with diabetes have a wide range of insulin requirements, bolus patterns, dietary habits, exercise habits, and many other lifestyle considerations that affect glycemic control. This means that these data should be interpreted with caution and should not be considered formulaic for clinical decision-making. The results may be used to supplement clinical heuristics that HCPs and users of CIQ already use when tuning insulin doses to maximize safety as well as glycemic outcomes.

## Conclusion

Overall, CIQ can improve outcomes for people with diabetes, and strategic insulin dose adjustments can maximize these outcomes. This analysis provides new evidence for leveraging Correction Factor and C:I ratio changes to maximize TIR while minimizing risk for increased hypoglycemia exposure.

## Prior Presentation

Data has not been presented before manuscript submission.

## Supplementary Material

Supplemental data

Supplemental data

Supplemental data
