# Supplementary material for: Therapy Settings Associated with Optimal Outcomes for t:slim X2 with Control-IQ Technology in Real-World Clinical Care
Source: Diabetes Technol Ther. 2023 Nov 23;25(12):877–82. doi: 10.1089/dia.2023.0308 (PMC10698772; doi:10.1089/dia.2023.0308)
Supplement: Supplemental data [file Supp_TableS1.docx]

Table S1: Median and interquartile ranges for consensus CGM metrics by quartiles of Correction Factor (Corr Factor), C:I ratio, and basal rate fraction TDI-based indices respectively.

|  | Corr Factor <1580/TDI | Corr factor between 1580/TDI and 1948/TDI | Corr Factor between 1948/TDI and 2422/TDI | Corr Factor >2422/TDI |
| --- | --- | --- | --- | --- |
| Percent Time in Range 70-180 [%] | 79.08 [71.5 - 85.83] | 72.56 [64.84 - 79.53] | 68.44 [60.08 - 76.29] | 64.96 [55.4 - 73.37] |
| Percent Time Below 70mg/dL [%] | 1.06 [0.49 - 2.04] | 1.02 [0.48 - 1.92] | 0.96 [0.43 - 1.81] | 0.91 [0.41 - 1.79] |
| Percent Time Above 180mg/dL [%] | 19.4 [12.43 - 27.21] | 25.98 [18.64 - 33.89] | 30.2 [22.1 - 38.8] | 33.73 [25.01 - 43.66] |
| Percent Time Above 250mg/dL [%] | 2.71 [1.02 - 5.69] | 5.15 [2.42 - 9.32] | 7.07 [3.45 - 12.37] | 9 [4.61 - 15.94] |
| Percent Time Below 54mg/dL [%] | 0.15 [0.05 - 0.34] | 0.14 [0.05 - 0.35] | 0.14 [0.05 - 0.32] | 0.13 [0.05 - 0.32] |
| Mean CGM [mg/dL] | 135.5 [135.5 - 155.9] | 143.7 [143.7 - 165.5] | 148 [148 - 173.5] | 151.9 [151.9 - 181.1] |
| Coefficient of Variation CGM [%] | 27.58 [24.51 - 30.79] | 29.62 [26.72 - 32.45] | 30.51 [27.62 - 33.41] | 31.02 [28.21 - 34.16] |
|  | C:I <330/TDI | C:I between 330/TDI and 417/TDI | C:I between 417/TDI and 522/TDI | C:I >522/TDI |
| Percent Time in Range 70-180 [%] | 76.96 [68.75 - 84.12] | 72.75 [64.7 - 80.33] | 69.99 [61.43 - 77.45] | 65.53 [55.68 - 74.26] |
| Percent Time Below 70mg/dL [%] | 1.08 [0.5 - 2.05] | 1.06 [0.52 - 1.97] | 0.96 [0.45 - 1.86] | 0.85 [0.35 - 1.67] |
| Percent Time Above 180mg/dL [%] | 21.47 [14.24 - 29.81] | 25.81 [18.13 - 33.96] | 28.66 [20.69 - 37.46] | 33.18 [23.99 - 43.46] |
| Percent Time Above 250mg/dL [%] | 3.38 [1.36 - 7.14] | 5.09 [2.31 - 9.51] | 6.39 [3.08 - 11.61] | 8.43 [4.18 - 15.37] |
| Percent Time Below 54mg/dL [%] | 0.15 [0.05 - 0.34] | 0.15 [0.05 - 0.35] | 0.14 [0.05 - 0.33] | 0.12 [0.04 - 0.31] |
| Mean CGM [mg/dL] | 137.6 [137.6 - 159.5] | 142.6 [142.6 - 165.5] | 146.4 [146.4 - 171.1] | 150.8 [150.8 - 180.8] |
| Coefficient of Variation CGM [%] | 28.54 [25.28 - 31.88] | 29.75 [26.66 - 32.85] | 30.1 [27.24 - 33.09] | 30.4 [27.51 - 33.32] |
|  | Basal <39.4% TDI | Basal between 39.4% and 47.6% of TDI | Basal between 47.6% and 56.5% of TDI | Basal >56.5% TDI |
| Percent Time in Range 70-180 [%] | 66.44 [56.9 - 75] | 69.78 [60.84 - 77.71] | 72.58 [64.11 - 79.94] | 76.45 [68.4 - 83.81] |
| Percent Time Below 70mg/dL [%] | 0.9 [0.4 - 1.72] | 0.97 [0.44 - 1.83] | 0.95 [0.45 - 1.84] | 1.15 [0.53 - 2.16] |
| Percent Time Above 180mg/dL [%] | 32.3 [23.36 - 42] | 28.9 [20.67 - 38] | 25.98 [18.43 - 34.67] | 21.79 [14.25 - 30.25] |
| Percent Time Above 250mg/dL [%] | 8.23 [3.91 - 15.12] | 6.44 [3.01 - 11.78] | 5.04 [2.37 - 9.61] | 3.52 [1.42 - 7.25] |
| Percent Time Below 54mg/dL [%] | 0.13 [0.05 - 0.31] | 0.14 [0.05 - 0.34] | 0.13 [0.04 - 0.31] | 0.16 [0.05 - 0.38] |
| Mean CGM [mg/dL] | 150.3 [150.3 - 179.8] | 146.6 [146.6 - 172.1] | 143.1 [143.1 - 166.5] | 136.9 [136.9 - 159] |
| Coefficient of Variation CGM [%] | 30.41 [27.33 - 33.42] | 30.08 [27.08 - 33.13] | 29.5 [26.52 - 32.51] | 28.95 [25.72 - 32.2] |
