# Supplementary material for: Therapy Settings Associated with Optimal Outcomes for t:slim X2 with Control-IQ Technology in Real-World Clinical Care
Source: Diabetes Technol Ther. 2023 Nov 23;25(12):877–82. doi: 10.1089/dia.2023.0308 (PMC10698772; doi:10.1089/dia.2023.0308)
Supplement: Supplemental data [file Supp_TableS2.docx]

Table S2: Medians for consensus CGM metrics by zoomed in bins of Correction Factor, C:I ratio, and basal rate fraction TDI-based indices respectively.

|  | Correction Factor TDI-based index | | | | | | | | |
| --- | --- | --- | --- | --- | --- | --- | --- | --- | --- |
|  | 1200-1300 | 1300-1400 | 1400-1500 | 1500-1600 | 1600-1700 | 1700-1800 | 1800-1900 | 1900-2000 | 2000-2100 |
| Percent Time in Range 70-180 [%] | 79.5 | 78.63 | 76.71 | 76.21 | 74.1 | 72.7 | 71.7 | 69.82 | 69.02 |
| Percent Time Below 70mg/dL [%] | 1.09 | 1.13 | 1.01 | 1.05 | 1.04 | 1.03 | 1.02 | 0.93 | 0.99 |
| Percent Time Above 180mg/dL [%] | 18.59 | 20.03 | 21.61 | 22.14 | 24.44 | 26.08 | 26.89 | 28.81 | 29.62 |
| Percent Time Above 250mg/dL [%] | 2.53 | 2.75 | 3.41 | 3.59 | 4.46 | 5.17 | 5.71 | 6.52 | 6.83 |
| Percent Time Below 54mg/dL [%] | 0.15 | 0.16 | 0.14 | 0.14 | 0.15 | 0.15 | 0.14 | 0.13 | 0.15 |
| Mean CGM [mg/dL] | 135 | 135.6 | 139.1 | 140.5 | 141.9 | 143.7 | 144.6 | 146.9 | 147.6 |
| Coefficient of Variation CGM [%] | 27.39 | 27.7 | 28.15 | 28.56 | 29.14 | 29.7 | 29.84 | 30.18 | 30.35 |
|  |  | | |  |  |  |  |  |  |
|  | C:I TDI-based index | | | | | | | | |
|  | 150-200 | 200-250 | 250-300 | 300-350 | 350-400 | 400-450 | 450-500 | 500-550 | 550-600 |
| Percent Time in Range 70-180 [%] | 80.79 | 78.7 | 76.53 | 74.79 | 72.46 | 71.56 | 69.51 | 67.55 | 66.17 |
| Percent Time Below 70mg/dL [%] | 1.09 | 1.07 | 1.07 | 1.08 | 1.08 | 1.01 | 0.96 | 0.91 | 0.85 |
| Percent Time Above 180mg/dL [%] | 17.84 | 19.48 | 21.91 | 23.67 | 26.07 | 26.95 | 29.1 | 31.02 | 32.57 |
| Percent Time Above 250mg/dL [%] | 2.36 | 2.82 | 3.43 | 4.21 | 5.26 | 5.61 | 6.57 | 7.73 | 8.06 |
| Percent Time Below 54mg/dL [%] | 0.15 | 0.15 | 0.14 | 0.15 | 0.16 | 0.14 | 0.14 | 0.12 | 0.13 |
| Mean CGM [mg/dL] | 132.1 | 135.5 | 138.4 | 140.6 | 143 | 144.4 | 147 | 148.7 | 150.1 |
| Coefficient of Variation CGM [%] | 27.39 | 27.94 | 28.76 | 29.13 | 29.99 | 29.89 | 30.23 | 30.38 | 30.41 |
|  |  |  |  |  |  |  |  |  |  |
|  | Basal TDI-based index | | | | | | | | |
|  | 30%-35% | 35%-40% | 40%-45% | 45%-50% | 50%-55% | 55%-60% | 60%-65% | 65%-70% | 70%-75% |
| Percent Time in Range 70-180 [%] | 65.29 | 65.92 | 67.41 | 69.42 | 70.96 | 72.8 | 74.49 | 75.79 | 78.77 |
| Percent Time Below 70mg/dL [%] | 0.85 | 0.92 | 0.92 | 0.96 | 0.94 | 0.97 | 1.01 | 1.12 | 1.28 |
| Percent Time Above 180mg/dL [%] | 33.8 | 32.83 | 31.22 | 29.32 | 27.7 | 25.85 | 24.21 | 22.62 | 19.4 |
| Percent Time Above 250mg/dL [%] | 9.31 | 8.44 | 7.58 | 6.74 | 5.79 | 4.99 | 4.2 | 3.89 | 2.82 |
| Percent Time Below 54mg/dL [%] | 0.12 | 0.13 | 0.14 | 0.14 | 0.13 | 0.14 | 0.14 | 0.16 | 0.18 |
| Mean CGM [mg/dL] | 152.8 | 150.5 | 148.9 | 147 | 145 | 143.1 | 141.3 | 138.2 | 132.9 |
| Coefficient of Variation CGM [%] | 30.45 | 30.55 | 30.36 | 30.16 | 29.74 | 29.53 | 29.07 | 29.34 | 28.54 |
