# Supplementary material for: Therapy Settings Associated with Optimal Outcomes for t:slim X2 with Control-IQ Technology in Real-World Clinical Care
Source: Diabetes Technol Ther. 2023 Nov 23;25(12):877–82. doi: 10.1089/dia.2023.0308 (PMC10698772; doi:10.1089/dia.2023.0308)
Supplement: Supplemental data [file Supp_TableS3.docx]

Table S3: Linear regression models of CGM outcomes

| **Time Below 54mg/dL** | Importance | Unstandardized Coefficients |  | Standardized Coefficients | | | 95.0% Confidence Interval for B | |
| --- | --- | --- | --- | --- | --- | --- | --- | --- |
| R^2^=0.012 |  | B | Std. Error | Beta | t | Sig. | Lower Bound | Upper Bound |
| (Constant) | - | 0.29 | 0.02 |  | 13.48 | 0.00 | 0.25 | 0.33 |
| Age [y] | 49% | -1.76E-03 | 1.47E-04 | -0.09 | -11.98 | 0.00 | -2.05E-03 | -1.47E-03 |
| TDI [U/kg] | 12% | -0.07 | 0.01 | -0.05 | -5.92 | 0.00 | -0.10 | -0.05 |
| Number of Manual Bolus | 15% | 7.92E-03 | 1.21E-03 | 0.05 | 6.56 | 0.00 | 5.56E-03 | 1.03E-02 |
| CRrule | 2% | -5.22E-05 | 2.15E-05 | -0.02 | -2.42 | 0.02 | -9.44E-05 | -9.97E-06 |
| CFrule | 1% | -8.33E-06 | 4.81E-06 | -0.01 | -1.73 | 0.08 | -1.78E-05 | 1.10E-06 |
| Basalrule | 21% | 0.20 | 0.03 | 0.06 | 7.76 | 0.00 | 0.15 | 0.25 |
|  |  |  |  |  |  |  |  |  |
| **Time Below 70mg/dL** | Importance | Unstandardized Coefficients |  | Standardized Coefficients | | | 95.0% Confidence Interval for B | |
| R^2^=0.036 |  | B | Std. Error | Beta | t | Sig. | Lower Bound | Upper Bound |
| (Constant) | - | 1.30 | 0.07 |  | 17.57 | 0.00 | 1.16 | 1.45 |
| Age [y] | 25% | -7.29E-03 | 5.05E-04 | -0.11 | -14.41 | 7.25E-47 | -8.28E-03 | -6.30E-03 |
| TDI [U/kg] | 8% | -0.35 | 0.04 | -0.07 | -8.28 | 0.00 | -0.44 | -0.27 |
| Number of Manual Bolus | 32% | 6.65E-02 | 4.14E-03 | 0.12 | 16.05 | 0.00 | 5.84E-02 | 7.46E-02 |
| CRrule | 8% | -5.86E-04 | 7.40E-05 | -0.06 | -7.93 | 0.00 | -7.31E-04 | -4.41E-04 |
| CFrule | 1% | -4.78E-05 | 1.65E-05 | -0.02 | -2.90 | 0.00 | -8.02E-05 | -1.55E-05 |
| Basalrule | 26% | 1.27 | 0.09 | 0.11 | 14.54 | 0.00 | 1.10 | 1.44 |
|  |  |  |  |  |  |  |  |  |
| **Time in Range 70-180** | Importance | Unstandardized Coefficients |  | Standardized Coefficients | | | 95.0% Confidence Interval for B | |
| R^2^=0.424 |  | B | Std. Error | Beta | t | Sig. | Lower Bound | Upper Bound |
| (Constant) | - | 66.02 | 0.52 |  | 127.32 | 0.00 | 65.00 | 67.04 |
| Age [y] | 2% | 0.04 | 0.00 | 0.07 | 12.39 | 0.00 | 0.04 | 0.05 |
| TDI [U/kg] | 7% | -7.74 | 44.00 | -0.17 | -25.98 | 0.00 | -8.32 | -7.15 |
| Number of Manual Bolus | 44% | 1.85 | 0.03 | 0.36 | 64.11 | 0.00 | 1.80 | 1.91 |
| CRrule | 13% | -1.77E-02 | 5.16E-04 | -0.20 | -34.36 | 0.00 | -1.88E-02 | -1.67E-02 |
| CFrule | 12% | -3.91E-03 | 1.15E-04 | -0.21 | -33.90 | 0.00 | -4.13E-03 | -3.68E-03 |
| Basalrule | 22% | 27.85 | 0.61 | 0.26 | 45.69 | 0.00 | 26.65 | 29.04 |
|  |  |  |  |  |  |  |  |  |
|  |  |  |  |  |  |  |  |  |
|  |  |  |  |  |  |  |  |  |
|  |  |  |  |  |  |  |  |  |
| **Time Above 180mg/dL** | Importance | Unstandardized Coefficients |  | Standardized Coefficients | | | 95.0% Confidence Interval for B | |
| R^2^=0.420 |  | B | Std. Error | Beta | t | Sig. | Lower Bound | Upper Bound |
| (Constant) | - | 32.67 | 0.54 |  | 60.99 | 0.00 | 31.62 | 33.73 |
| Age [y] | 1% | -0.04 | 0.00 | -0.06 | -9.99 | 0.00 | -0.04 | -0.03 |
| TDI [U/kg] | 7% | 8.09 | 0.31 | 0.17 | 26.29 | 0.00 | 7.49 | 8.69 |
| Number of Manual Bolus | 44% | -1.92 | 0.03 | -0.36 | -64.28 | 0.00 | -1.98 | -1.86 |
| CRrule | 13% | 1.83E-02 | 5.34E-04 | 0.20 | 34.36 | 0.00 | 1.73E-02 | 1.94E-02 |
| CFrule | 12% | 3.95E-03 | 1.19E-04 | 0.20 | 33.21 | 0.00 | 3.72E-03 | 4.19E-03 |
| Basalrule | 23% | -29.11 | 0.63 | -0.27 | -46.24 | 0.00 | -30.35 | -27.88 |
|  |  |  |  |  |  |  |  |  |
| **Time Above 250mg/dL** | Importance | Unstandardized Coefficients |  | Standardized Coefficients | | | 95.0% Confidence Interval for B | |
| R^2^=0.383 |  | B | Std. Error | Beta | t | Sig. | Lower Bound | Upper Bound |
| (Constant) | - | 11.94 | 0.32 |  | 37.52 | 0.00 | 11.32 | 12.56 |
| Age [y] | 8% | -0.05 | 0.00 | -0.15 | -23.89 | 0.00 | -0.06 | -0.05 |
| TDI [U/kg] | 7% | 4.14 | 0.18 | 0.15 | 22.67 | 0.00 | 3.79 | 4.50 |
| Number of Manual Bolus | 46% | -1.05 | 0.02 | -0.34 | -59.13 | 0.00 | -1.08 | -1.01 |
| CRrule | 10% | 8.84E-03 | 3.17E-04 | 0.17 | 27.89 | 0.00 | 8.22E-03 | 9.46E-03 |
| CFrule | 10% | 1.93E-03 | 7.07E-05 | 0.17 | 27.27 | 0.00 | 1.79E-03 | 2.07E-03 |
| Basalrule | 19% | -14.30 | 0.37 | -0.23 | -38.23 | 0.00 | -15.03 | -13.57 |
|  |  |  |  |  |  |  |  |  |
| **Mean CGM** | Importance | Unstandardized Coefficients |  | Standardized Coefficients | | | 95.0% Confidence Interval for B | |
| R^2^=0.413 |  | B | Std. Error | Beta | t | Sig. | Lower Bound | Upper Bound |
| (Constant) | - | 167.53 | 0.87 |  | 193.52 | 0.00 | 165.84 | 169.23 |
| Age [y] | 0% | -0.04 | 0.01 | -0.04 | -6.43 | 0.00 | -0.05 | -0.03 |
| TDI [U/kg] | 7% | 13.03 | 0.50 | 0.17 | 26.21 | 0.00 | 12.06 | 14.01 |
| Number of Manual Bolus | 43% | -3.10 | 0.05 | -0.36 | -64.20 | 0.00 | -3.19 | -3.01 |
| CRrule | 12% | 2.97E-02 | 8.62E-04 | 0.21 | 34.41 | 0.00 | 2.80E-02 | 3.14E-02 |
| CFrule | 10% | 5.81E-03 | 1.92E-04 | 0.19 | 30.20 | 0.00 | 5.43E-03 | 6.19E-03 |
| Basalrule | 27% | -51.30 | 1.02 | -0.29 | -50.42 | 0.00 | -53.30 | -49.31 |
|  |  |  |  |  |  |  |  |  |
|  |  |  |  |  |  |  |  |  |
|  |  |  |  |  |  |  |  |  |
|  |  |  |  |  |  |  |  |  |
| **Coefficient of Variation** | Importance | Unstandardized Coefficients |  | Standardized Coefficients | | | 95.0% Confidence Interval for B | |
| R^2^=0.208 |  | B | Std. Error | Beta | t | Sig. | Lower Bound | Upper Bound |
| (Constant) | - | 33.47 | 0.22 |  | 149.33 | 0.00 | 33.03 | 33.91 |
| Age [y] | 48% | -0.06 | 0.00 | -0.30 | -42.38 | 0.00 | -0.07 | -0.06 |
| TDI [U/kg] | 0% | -0.35 | 0.13 | -0.02 | -2.70 | 0.01 | -0.60 | -0.10 |
| Number of Manual Bolus | 35% | -0.45 | 0.01 | -0.24 | -36.10 | 0.00 | -0.48 | -0.43 |
| CRrule | 1% | 1.10E-03 | 2.23E-04 | 0.03 | 4.93 | 0.00 | 6.63E-04 | 1.54E-03 |
| CFrule | 14% | 1.12E-03 | 4.98E-05 | 0.16 | 22.46 | 0.00 | 1.02E-03 | 1.22E-03 |
| Basalrule | 2% | -2.32 | 0.26 | -0.06 | -8.82 | 0.00 | -2.84 | -1.81 |
